# Psychosocial Rehabilitation Through Intervention by Second Language Acquisition in Older Adults

**DOI:** 10.1007/s10936-021-09805-z

**Published:** 2021-08-11

**Authors:** Marcel Pikhart, Blanka Klimova, Anna Cierniak-Emerych, Szymon Dziuba

**Affiliations:** 1 Department of Applied Linguistics, Faculty of Informatics and Management, University of Hradec Kralove, Hradec Kralove, Czech Republic; 2 Faculty of Business and Management, Wroclaw University of Economics and Business, ul. Komandorska 118/120, 53-345 Wrocław, Poland

**Keywords:** Foreign language learning, Psycholinguistics, FLL, L2 acquisition, Seniors, Wellbeing, Aging, Cognitive decline, Successful aging, Healthy aging

## Abstract

The paper deals with a possibility of foreign language learning (FLL) intervention in older adults as a psychosocial rehabilitation method to improve the quality of life (QoL) in this age segment, i.e. the people who are over 55 years. FLL has been researched as a successful tool to maintain or even enhance cognitive functions in older age along with other intentional activities, such as engagement in any physical activity. FLL cannot dramatically improve memory and cognitive deterioration of older adults, however, it can improve QoL by increasing subjective happiness that is connected to improved wellbeing. The research was conducted in two groups of seniors who are engaged in FLL, specifically in the Czech Republic and Poland. The major premise of the research was based on the positive psychology concept, i.e., the subjective happiness leads to improved levels of QoL. Both groups consisted of about a hundred respondents whose opinions were researched by an online questionnaire. The major focus of this questionnaire was to evaluate the level of subjective happiness and then compare the results obtained from the respondents from these two geographically different regions. The findings clearly showed that those who had engaged in FLL had reached high levels of subjective happiness, therefore, their subjective wellbeing could be improved. These results might be important for psychosocial rehabilitation practices because they can create a framework for further non-pharmacological intervention to maintain healthy aging. FLL can thus be a very efficient tool for any psychosocial rehabilitation in older healthy adults who do not suffer from any cognitive pathological development but who are just experiencing negative side effects of natural aging process. The research into this topic is very scarce, and therefore, this paper could be an inspiration for further and larger-scale research.

## Introduction

The research into various aspects of foreign language learning (FLL) is ample, however, there is a significant impact of FLL on maintaining or even enhancing cognitive parameters in older adults as well (Klimova, 2018; Klimova et al., 2016; Phenniger et al., 2018; Pikhart & Klimova, 2020; Valis et al., 2019). The natural aging process is connected to many psychological and mental aspects and one of them is the process of cognitive decline, well documented by numerous research (Murman, 2015; Salthouse, 2010). It is an inevitable process connected to aging, however, there are various activities that could have a potential to slow down this natural process, such as music, natural environment, nutrition, physical activity, mental activity, arts, or political and civic engagement (Cann, 2017; Elzen et al., 2019; Erickson et al., 2009; Gil-Lacruz et al., 2020; Klimova et al., 2020; MacKean et al., 2011; Mathis et al., 2016; Socci et al., 2020; Serrat et al. 2019, Serrat et al., 2015; Wilson et al., 2006). These non-pharmacological intervention activities work very well in various psychological perspectives and it is very clear that they improve objective physical and mental status, and consequently, also subjective wellbeing of the participants of these activities.

Quality of life (QoL) is connected to objective parameters, such as healthy lifestyle, physical activity, or social contacts, but also to subjective parameters, and these are very much associated with subjective–therefore, difficult to measure–feelings of happiness. The basic premise of this paper is that QoL is significantly influenced by subjective feelings of happiness. If it is so, how much FLL can improve these subjective feelings of happiness and thus lead to improved subjective wellbeing and hence QoL? There is a clear correlation between subjective feelings of satisfaction and happiness. Moreover, happiness correlates with improved QoL.

FLL has not much been associated with happiness and wellbeing but rather on the contrary. Learning psychology maintains that schooling and education can lead to the systematic oppression of creativity and in some cases, even to depression (Zhang et al., 2020). However, recent research into FLL and psycholinguistics has shown (Pikhart & Klimova, 2020) that in older generation FLL can have a very different impact on the personality through improved subjective feelings of satisfaction. In younger adults, the research results indicate that despite their relatively good study results and learning outcomes in FLL or second language (L2) acquisition, they do not reach very high levels in their subjective satisfaction and feelings of wellbeing connected to their FLL. These younger learners see their FLL as a pragmatic activity leading to certain results, necessary for their work and future prospects of travelling and earning money. They do not experience any elevated levels of happiness arising from their FLL per se. The learning activity is a must for them because it brings monetary benefits and a future competitive advantage (Kormos & Csizer, 2008). On the contrary, in older adults–despite their slightly worse objective learning results in their L2 acquisition–they usually express their inner subjective satisfaction with their FLL regardless of their not very motivating objective outcomes. This goes in opposition with traditional learning psychology theory, i.e., the good learning results are a motivator and the poor learning results are a demotivator. Counterintuitively, in a generational comparison, it does not work so and this assertion is not applicable.

Moreover, FLL can be used as a tool for psychosocial rehabilitation because the learning process always works as a cognitively-mental tool and FLL is usually a social activity, at least in the case of class learning. Moreover, FLL can also be one of the non-pharmacological tools to slow down the onset of serious neurodegenerative diseases, as research has recently confirmed (Antoniou et al., 2017; Bubbico et al., 2019; Klimova et al., 2016). Psychosocial rehabilitation through FLL can, therefore, have a potential to be a relatively useful and cheap method to enhance subjective wellbeing of participants through the intervention by cognitive training, and societies can benefit from this by saving costs on pharmacological interventions in serious cognitive illnesses whose onset can be thus delayed (Ware et al., 2017; Wong et al., 2019).

The research question was as follows: How much will the level of subjective wellbeing be influenced by the participation in FLL in older adults? If the level of wellbeing reaches very high levels, it is possible to come to a conclusion that FLL improves significantly the wellbeing levels and therefore can be very efficient tool for psychosocial rehabilitation in older age.

## Methodology

The research sample included two groups of older individuals at the age of 55 + years from two neighboring Central European countries, specifically, Poland and the Czech Republic. The Czech sample consisted of 105 participants and the Polish sample comprised 100 participants. All were either attendees of the University of the Third Age (U3A) or studied a foreign language privately. The respondents did not suffer from any serious cognitive or mental impairments, such as dementia or Alzheimer´s disease.

The participants (randomly selected by e-mail contacts) were asked to fill in an online questionnaire via Google Forms. The questionnaire included 20 statements concerning elderly people’s attitude to FLL from the viewpoint of positive psychology, as well as four questions on their demographic data. The questionnaire in a slightly modified version following a standardized questionnaire designed by Woll &Wei (2019). The participants provided their responses on the 6-point Likert scale (strongly agree, agree, agree a little, disagree a little, disagree, and strongly disagree). The collection of data was conducted between April 2020 and June 2020.

The research complies with the Helsinki declaration because all participants were informed about the purpose of the study, and about the possibility of withdrawing from participation in the research at any time, as well as giving informed and voluntary consent to participate in this research. The intervention by FLL, i.e. the participation in the FLL class, had been approved by the written consent of all participants before the course started. All the regulations demanded by the EU regulation GDPR were followed, i.e. no personal data about the participants was collected or stored. The only identification of the questionnaire was the data stamp of the questionnaire that does not contain any other information but the date and time of the data collection. No IP addresses were collected either. This research was approved by the Ethics Committee of the University of Hradec Kralove (no. 2/2021).

The paper used the methods of descriptive statistics and parametric and non-parametric significance tests of differences between independent groups as they could easily reveal the answer to the research question and clearly indicate the relevance of FLL and its impact on the subjective feelings of happiness. Furthermore, reliability coefficients were calculated for the scales created, based on the modified Q-Sort method for subject-related data sorting. In addition, multidimensional regression analyses were performed to determine which demographic variables would affect the assessment of language learning at individual scales among people of both nationalities. The calculations were performed using the PQ Stat software, ver. 18.0. The statistical significance was set at *p* = 0.05.

## Results

The research tool was a list of twenty statements covering various advantages and disadvantages of FLL in terms of mental wellbeing. The research result analysis uses selected statements relating to the advantages of language learning. The participants responded on a scale of 1 to 6 points (6—I absolutely agree, 5—I agree, 4—I agree partially, to 1—I absolutely disagree). The answers were grouped into three categories (I agree, I disagree, I am not sure) for legibility of interpretation (Table 1). Of all respondents, 94% provided complete answers that were used in the survey (100% PL, 88% CZ).

**Table 1:** Descriptive statistics for statements assessed on a scale of 1 to 6 (1- I absolutely disagree, 6- I absolutely agree)

Statement	Country	M	SD	Frequency (%)	
				I agree (I absolutely agree, I agree, I rather agree)	I disagree (I absolutely disagree, I disagree, I rather disagree)
1. Learning a new language improves my concentration	CZ	4.65	0.99	98.9	1.1
	PL	5.38	0.76	99.0	1.0
2. Learning a new language improves my memory	CZ	4.80	1.06	98.9	1.1
	PL	5.36	0.77	96.0	4.0
3. Learning a new language improves my attention	CZ	4.69	0.96	100.0	0.0
	PL	5.20	0.90	95.0	5.0
4. Learning a new language improves my health	CZ	4.13	0.94	84.8	15.2
	PL	4.38	1.12	77.0	23.0
5. Learning a new language improves my creativity	CZ	4.42	0.99	92.4	7.6
	PL	5.16	0.84	95.0	5.0
6. Learning a new language helps me find new friends	CZ	4.70	0.99	97.8	2.2
	PL	5.22	0.76	100.0	0.0
7. Learning a new language helps me understand different cultures	CZ	4.93	1.05	98.9	1.1
	PL	5.27	0.75	98.0	2.0
8. Learning a new language helps me while travelling	CZ	5.26	1.05	95.7	4.3
	PL	5.49	0.80	98.0	2.0
9. Learning a new language helps me with learning other things, too	CZ	4.49	0.92	96.8	3.2
	PL	5.13	0.80	97.0	3.0
10. Learning a new language helps me while looking for life motivation	CZ	4.50	1.04	94.6	5.4
	PL	4.67	1.03	88.0	12.0
11. Learning a new language helps me with finding the purpose of my life	CZ	3.97	1.09	81.5	18.5
	PL	4.45	1.23	83.0	17.0
12. Learning a new language is enjoyable	CZ	4.84	1.09	96.7	3.3
	PL	5.05	1.05	94.0	6.0
13. Learning a new language brings me personal satisfaction	CZ	4.73	1.14	96.7	3.3
	PL	5.31	0.79	97.0	3.0
14. Learning a foreign language brings me feelings of happiness	CZ	4.72	1.13	94.6	5.4
	PL	5.10	1.03	95.0	5.0
15. Learning a new language is a positive motivation for me	CZ	4.63	1.11	94.6	5.4
	PL	5.18	0.94	96.0	4.0
16. Learning a new language will be useful for me in the future	CZ	4.63	1.13	92.4	7.6
	PL	5.18	0.98	96.0	4.0

*M*, mean; *SD*, tandard deviation

Table 1 contains average values and standard deviations for individual items (statements) concerning the positive effects of language learning. The vast majority of respondents from both countries agreed with the statements. Differences in mean assessments between the Czech and Polish seniors are therefore largely due to more frequent choices of more unequivocal positive replies by the Polish respondents (I absolutely agree, I agree) compared to the Czech respondents (e.g. statements 1, 9, 11, 13). Three statements with the greatest differences between countries in the frequency of agreement and disagreement were distinguished. These are the statements number 3, 4 and 10 in Table 1, they are also highlighted in bold letters.

Individual statements were categorized into four thematic groups (scales), based on their content, i.e. intellect, travelling and future, positive emotions, and purpose and motivation. Table 2 contains sets of statements on each scale and reliability coefficients. The quality of scales measured by reliability was good or very good in the Czech study (Cronbach’s α coefficient from 0.78 to 0.93) and slightly lower in the Polish study (Cronbach's α coefficient from 0.72 to 0.91). The scale of *Positive emotions* was the most consistent, whereas the least consistent was the scale of *Travelling and future.* The statement 5 (“Learning a new language improves my creativity”) was excluded during analysis as it is inconsistent with other statements (taking it into account on the Intellect scale significantly reduced the α coefficient for each country).

**Table 2:** Statements in individual scales and reliability of the scales

Scale	Items	Scale reliability (Cronbach’s α)	
		CZ	PL
Intellect	1. Learning a new language improves my concentration 2. Learning a new language improves my memory 3. Learning a new language improves my attention 9. Learning a new language helps me with learning other things, too	0.86	0.85
Travelling and future	8. Learning a new language helps me while travelling 7. Learning a new language helps me understand different cultures 20. Learning a new language will be useful for me in the future	0.78	0.72
Positive emotions	12. Learning a new language is enjoyable 13. Learning a new language brings me personal satisfaction 14. Learning a foreign language brings me feelings of happiness	0.93	0.91
Purpose and motivation	10. Learning a new language helps me while looking for life motivation 11. Learning a new language helps me with finding the purpose of my life 19. Learning a new language is a positive motivation for me	0.81	0.72

The Poles assessed the positive effects of FLL significantly higher than the Czechs in every aspect: intellectual (*p* < 0.001), travelling and future (*p* < 0.01), positive emotions (*p* < 0.01) and life purpose and motivation (*p* < 0.01), see Table 3.

**Table 3:** Mean assessments of the Czechs and Poles on the scales of the effects of language learning and the significance of differences between the groups

Scale	CZ (*n* = 92)		PL (*n* = 100)		Significance of differences
	M	SD	M	SD	
Intellect	4.7	0.8	5.3	0.7	*U* = 2708, *p* < 0.001**
Travelling and future	4.9	0.9	5.3	5.3	*U* = 3653,5 *p* = 0.006*
Positive emotions	4.8	1.0	5.1	0.9	*t* (190) = − 2.82, *p* = 0.005*
Purpose and motivation	4.4	0.9	4.8	0.9	*t* (190) = − 3.11, *p* = 0.002*

*statistically significant difference at *p* < 0.01 **statistically significant difference at *p* < 0.01

The statistical analysis of the mean assessments of the effects of FLL on mental wellbeing considered the following demographic variables: **age, gender, level of education, number of languages** learnt at the time of the survey and the currently learnt language.

**Age**: Comparison of peers from Poland and the Czech Republic revealed that wherever significant differences had occurred, the mean higher assessments had been always given by the Polish respondents (Table 4).

**Table 4:** Mean assessments of age groups of the Czechs and Poles on the scales of effects of language learning and the significance of differences

Scale	55–60 years			61–65 years			66–70 years		
	CZ (*n* = 16) (MSD)	PL (*n* = 62) (MSD)	Significance of differences	CZ (*n* = 21) (MSD)	PL (*n* = 28) (MSD)	Significance of differences	CZ (*n* = 17) (MSD)	PL (*n* = 7) (MSD)	Significance of differences
Intellect	5.1 1.0	5.3 0.7	*U* = 494.5 *p* = 0.99	4.5 0.7	5.3 0.6	*t* (47) = − 3.78 *p* < 0.001***	4.4 0.6	5.4 0.5	*U* = 14 *p* = 0.001**
Travelling and future	5.5 0.9	5.4 0.6	*U* = 260.5 *p* = 0.08	4.8 0.8	5.1 0.8	*t* (47) = − 1.10 *p* = 0.27	4.3 0.7	5.3 0.4	*U* = 6.5 *p* < 0.001***
Positive emotions	5.3 0.9	5.1 0.9	*U* = 426.5 *p* = 0.38	4.7 1.0	5.1 0.8	*t* (47) = − 1.77 *p* = 0.08	4.4 0.8	5.4 0.5	*U* = 21.5 *p* = 0.009**
Purpose and motivation	4.9 1.0	4.7 0.9	*U* = 442.5 *p* = 0.51	4.1 0.8	4.8 0.8	*t* (47) = − 3.08 *p* = 0.01*	4.1 0.8	4.9 0.6	*U* = 22 *p* = 0.01*

*statistically significant difference at *p* < 0.05, **statistically significant difference at *p* < 0.01, ***statistically significant difference at *p* < 0.001

**Gender**: The analysis of the differences in the perceived effects of FLL on wellbeing between the genders (male and female) revealed the following statistically significant relationships: The Polish females evaluated the positive effects of FLL significantly higher than the Czech females in every aspect: intellectual (*p* < 0.001), travelling and future (*p* < 0.001), positive emotions (*p* < 0.001) and life purpose and motivation (*p* < 0.001). On the contrary, there were no statistically significant differences in the assessment of individual dimensions of the quality of life between the males from both countries (Table 5).

**Table 5:** Mean assessments of women and men from the Czech Republic and Poland on the scales of effects of language learning and the significance of differences

Scale	Men			Women		
	CZ (*n* = 33) (MSD)	PL (*n* = 40) (MSD)	Significance of differences	CZ (*n* = 59) (MSD)	PL(*n* = 60) (MSD)	Significance of differences
Intellect	4.8 0.8	5.1 0.6	*t* (56) = − 1.98 *p* = 0.05	4.6 0.8	5.4 0.7	*t* (117) = − 5.52 *p* < 0.001*
Travelling and future	5.2 0.8	5.2 0.6	*t* (71) = 0.14 *p* = 0.88	4.8 0.8	5.4 0.7	*t* (117) = − 4.08 p < 0.001*
Positive emotions	5.1 1.0	5.0 0.8	*t* (71) = 0.05 *p* = 0.96	4.6 1.0	5.2 0.9	*t* (117) = − 3.49 p < 0.001*
Purpose and motivation	4.6 0.8	4.6 0.7	*t* (71) = 0.05 *p* = 0.64	4.2 0.9	4.8 0.9	*t* (117) = − 3.39 *p* < 0.001*

*Statistically significant difference at *p* < 0.001

Place of residence: The comparative analysis showed that there were no significant differences in the assessment of positive effects of FLL between the inhabitants of the cities and the inhabitants of rural areas from both countries (Table 6).

**Table 6:** Mean assessments of people living in rural areas and cities from the Czech Republic and Poland on the scales of the effects of language learning and the significance of differences

Subscale	Inhabitants of cities			Inhabitants of rural areas		
	CZ (*n* = 63) (MSD)	PL (*n* = 84) (MSD)	Significance of differences	CZ (*n* = 29) (MSD)	PL (*n* = 16) (MSD)	Significance of differences
Intellect	5.1 0.8	4.8 0.8	*U* = 2233 *p* = 0.10	5.0 0.8	5.2 0.9	*U* = 191 *p* = 0.33
Travelling and future	5.2 0.7	5.1 0.8	*U* = 2596 *p* = 0.84	5.1 1.0	5.4 0.8	*U* = 177.5 *p* = 0.18
Positive emotions	5.0 1.0	4.8 1.0	*U* = 2408.5 *p* = 0.34	5.0 0.9	5.3 0.9	*U* = 177.5 *p* = 0.17
Purpose and motivation	4.5 0.9	4.5 0.9	*U* = 2597.5 *p* = 0.85	4.7 0.9	4.9 0.7	*U* = 192 *p* = 0.34

Education: In the group of people without higher education (in the Czech Republic, these were people with secondary education, whereas in Poland—people with primary and vocational education), the Czechs assessed the increase in positive emotions caused by learning a foreign language higher than the Poles. In other assessments, the inhabitants of Poland and the Czech Republic with a comparable level of education did not differ from each other (Table 7).

**Table 7:** Mean assessments in groups of education of the Czechs and Poles on the scales of effects of language learning and significance of differences

Subscale	Secondary and lower education			Higher education		
	CZ (*n* = 38) (MSD)	PL (*n* = 8) (MSD)	Significance of differences	CZ (*n* = 54) (MSD)	PL (*n* = 92) (MSD)	Significance of differences
Intellect	5.1 0.7	4.7 0.8	*U* = 108 *p* = 0.20	5.0 0.8	4.9 0.8	*U* = 2352.5 *p* = 0.59
Travelling and future	5.2 0.6	5.1 0.8	*U* = 136 *p* = 0.65	5.1 0.8	5.1 0.8	*U* = 2336 *p* = 0.54
Positive emotions	5.1 0.8	4.1 1.5	*U* = 82.5 *p* = 0.04*	4.9 1.0	5.0 0.9	*U* = 2391.5 *p* = 0.70
Purpose and motivation	4.7 0.8	4.4 1.4	*U* = 139 *p* = 0.72	4.5 0.9	4.6 0.9	*U* = 2240 *p* = 0.32

*Statistically significant difference at p < 0.05

Number of languages: At the time of the survey, there were 67.4% Czech learners and 70.0% Polish learners of one foreign language, 17.4% Czechs and 23.0% Poles learning two languages, and 15.2% of Czechs and 7.0% of Poles learning three languages. There were no differences in the assessment of the positive effects of language learning between the groups of the Czechs and Poles learning the same number of languages (Table 8).

**Table 8:** Mean assessments of the effects of language learning in groups of learners of different numbers of languages and the significance of differences

Subscale	One			Two			Three or more		
	CZ *n* = 62 (MSD)	PL *n* = 70 (MSD)	Significance of differences	CZ *n* = 16 (MSD)	PL *n* = 23 (MSD)	Significance of differences	CZ *n* = 14 (MSD)	PL *n* = 7 (MSD)	Significance of differences
Intellect	4.9 0.8	4.7 0.8	*t* (130) = 1.16 *p* = 0.25	5.3 0.6	5.3 0.6	*U* = 176 *p* = 0.83	4.3 0.6	5.3 0.6	*U* = 48.5 *p* = 0.99
Travelling and future	5.0 0.8	4.9 0.9	*t* (130) = -0.02 *p* = 0.98	5.5 0.5	5.6 0.5	*U* = 166.5 *p* = 0.62	5.0 0.6	5.4 0.6	*U* = 45 *p* = 0.79
Positive emotions	4.9 1.0	4.8 1.1	*t* (130) = 0.66 *p* = 0.51	5.3 0.8	5.3 0.9	*U* = 426.5 *p* = 0.38	4.3 1.2	5.2 0.7	*U* = 169.5 *p* = 0.67
Purpose and motivation	4.5 0.9	4.4 0.9	*t* (130) = 0.39 *p* = 0.69	4.2 0.8	4.9 0.8	*U* = 163.5 *p* = 0.56	3.9 0.7	5.0 0.6	*U* = 31.5 *p* = 0.20

In order to identify the determinants of the assessment of different aspects of language learning, four independent logistic regression analyses were performed (for the scales of Intellect, Travelling and future, Positive emotions, Purpose and motivation) for each nationality. In each of these analyses, diverse demographic variables were used as predictors in the model. The basic models for each scale included all demographic variables from the survey: gender, age, level of education, place of residence, number of languages the respondent learnt at the time of the survey.

### Intellect Scale

Czechs: In the model for the *Intellect scale*, the results show that an important predictor of the assessment of the effect of language learning on intellect is the number of languages learnt (*β* = 0.27, *t* = 2.53, *p* = 0.01). The significant model is as follows: F (3.90) = 6.40, *p* = 0.01, and explains 6.6% of the variance (corrected *R*^2^ = 0.056). The results mean that individuals who learn more languages will be more likely to see positive effects of language learning in the area of intellect.

Poles: In the model for the Intellect scale, the results showed that an important predictor of the assessment of the effect of language learning on intellect are gender (*β* = − 0.42, *t* = − 2.52, *p* = 0.01) and the number of languages (*β* = 0.38, *t* = 3.08, *p* = 0.003). Gender explains 6.1% of the variance (corrected *R*^2^ = 0.051), whereas the number of languages explains 8.8% of the variance (corrected *R*^2^ = 0.078). The results obtained mean that being a woman and the greater number of languages learnt increase the assessment of the positive effects of FLL in terms of intellectual abilities.

### Scale of Travelling and future

Czechs: In the model for the scale of *Travelling and future*, the results show that an important predictor of the assessment of the effect of FLL on travelling and better future is the number of languages learnt (*β* = 0.28, *t* = 2.69, *p* = 0.008). The significant model is as follows: F (3.90) = 7.22, *p* = 0.01 and explains 7.4% of the variance (corrected *R*^2^ = 0.064). The results mean that individuals who learn more languages will be more likely to see positive effects of language learning in the area of travelling and future.

**Poles**: In the model for the scale of Travelling and future, the results show that an important predictor of the assessment of the effect of language learning on travelling and better future is the number of languages learnt (*β* = 0.32, *t* = 2.52, *p* = 0.01). The significant model is as follows: F (3.98) = 6.37, *p* = 0.01 and explains 6.1% of the variance (corrected *R*^2^ = 0.051). The results mean that individuals who learn more languages will be more likely to see positive effects of language learning in the area of travelling and future.

### Scale of Positive Emotions

Czechs: In the model for the scale of positive emotions, none of the predictors was statistically significant.

Poles: In the model for the scale of positive emotions, the results showed that an important predictor of the assessment of the effect of language learning on positive emotions are gender (*β* = − 0.48, *t* = − 2.33, *p* = 0.02) level of education (*β* = 0.87, *t* = − 2.33, *p* = 0.02), and the number of languages (*β* = 0.34, *t* = 2.15, *p* = 0.03). Gender explains 5.2% of the variance (corrected *R*^2^ = 0.043), level of education explains 4% of the variance (corrected *R*^2^ = 0.054), and the number of languages explains 3% of the variance (corrected *R*^2^ = 0.045). The results obtained mean that being a woman, having a higher level of education, and the greater number of languages learnt increased the assessment of the positive effects of language learning on the scale of positive emotions.

### Scale of Purpose and Motivation

Czechs: In the model for the scale of purpose and motivation, none of the predictors was statistically significant.

Poles: In the model for the scale of purpose and motivation, the results show that an important predictor of the assessment of the effect of language learning on purpose and motivation is the number of languages learnt (*β* = 0.36, *t* = 2.54, *p* = 0.01). The significant model is as follows: F (3.98) = 6.47, *p* = 0.01 and explains 6.2% of the variance (corrected *R*^2^ = 0.052). The results mean that individuals who learn more languages will be more likely to see positive effects of language learning in the area of purpose and motivation.

## Discussion

The findings of this study reveal that the questionnaire statements on the basis of their meaning can be divided into four thematic groups: *Intellect, Travelling and future, Positive emotions*, and *Purpose and motivation*. The most consistent variable in both groups (Czech and Polish) were *Positive emotions*, which confirms our assumption that elderly people study a foreign language because it brings them happiness and personal satisfaction, which, consequently, positively affects their overall wellbeing (Pikhart & Klimova, 2020). In addition, the findings show that the Polish respondents assessed the positive effects of FLL in all four thematic categories significantly higher than the Czech respondents. This might be associated with the fact that the number of the Poles with university education was higher than in the group of the Czech seniors. For instance, Belo et al. (2020) in their study report that seniors with high education and a more positive leisure attitude have a better psychological adjustment concerning wellbeing. Moreover, the Polish females evaluated the positive effects of FLL considerably higher than the Czech women. However, this discrepancy does not create any obstacle in concluding that in both groups of respondents the overall wellbeing levels obtained by FLL were very high, and this intervention by L2 acquisition can function as an efficient tool to improve QoL.

The results also reveal that the Czech seniors with secondary education compared to the Polish seniors with primary and vocational education assessed the increase in positive emotions caused by learning a foreign language higher than the Poles. As for other demographic factors (age, place of residence and number of languages) and their correlation to the positive effects of FLL, the findings do not show any significant differences in both experimental groups.

In addition, the results of the logistic regression analyses in the Czech group show that the more languages a senior learns, the more positive effects of FLL s/he perceives in the area of intellect. In the Polish group, this concerns only females. This is also evidenced by research studies on FLL and cognition and bilingualism, which imply that people who have a command of another foreign language or start learning it in older age may improve their cognitive skills (Antoniou, 2019; Bialystok, 2017; Bubbico et al., 2019; Wong et al., 2019).

Furthermore, motivation for older people to study a foreign language is their desire to travel, especially in the countries which were for a long time behind the iron curtain and people were not allowed to travel, i.e. both the Czech Republic and Poland. Moreover, those who can master more language are also more self-confident in encountering foreign cultures. Therefore, the results also indicate that individuals in both groups who learn more languages will be more likely to see positive effects of FLL in the area of travelling and future outlooks despite the age limitation of the participants of the study. The more language a Polish senior learns also indicates that s/he will more likely see positive effects of language learning in the area of purpose and motivation.

Naturally, there are several limitations to this study. First, the research sample is from two Central European countries, therefore, it is necessary to validate these findings on a larger scale. However, the research sample was representative enough and this limitation does not pose any threat to the reliability of the findings. Ideally, it would be beneficial to conduct similar research globally, or at least in several continents, to increase the statistical reliability. Second, the research was conducted in seniors, i.e. those who are older than 55 years. It would be interesting to focus only on a group of elderly people, e.g. older than 70 years, and observe how much L2 acquisition can help this cohort regarding their psychosocial well-being. However, it is extremely complicated to find a research sample large enough with these parameters, i.e. older than 70 and taking part in foreign language learning. But these parameters could also indicate future lines of research. Moreover, it seems important to determine what the cognitive mechanisms are responsible for this high level of subjective satisfaction and well-being.

The implications of our findings are both for the theory and practice. For the theory of psycholinguistics, these findings are crucial as there is no relevant or similar research into the topic we analyzed. Therefore, our findings have to be verified by on a large sale so as to provide psycholinguistic theory with relevant theoretical concepts regarding L2 acquisition in an older age. As we have already noted, the topic of L2 acquisition in an older age is still generally missing in psycholinguistic textbooks despite the fact that it is crucially beneficial and thus important not to be neglected or ignored. The implications for the practice result from the fact that L2 acquisition in an older age significantly improves the QoL and thus the well-being of the participant is enhanced, too. Therefore, FLL can be one of the intervention strategies, which can contribute to overall well-being among cognitively healthy seniors.

The research conducted clearly indicated that FLL or L2 acquisition in an older age can significantly improve the QoL in the participants, and that this improved level of wellbeing can function as a tool of psychosocial rehabilitation. The fact that the seniors take part in a cognitively stimulation environment and simultaneously they meet with their peers has a very positive effect on their increased sense of achievement, sense of life purpose and future prospects. FLL as an interventional method can function very well as a non-pharmacological tool, and if supplemented by physical activity, games, music, art, improved diet, etc., it can create a very prospective tool for psychosocial rehabilitation of seniors.

## Declarations

### Data availability

Upon request from the corresponding author.
